# The oncogenic mutation in the pleckstrin homology domain of AKT1 in endometrial carcinomas

**DOI:** 10.1038/sj.bjc.6605109

**Published:** 2009-06-02

**Authors:** K Shoji, K Oda, S Nakagawa, S Hosokawa, G Nagae, Y Uehara, K Sone, Y Miyamoto, H Hiraike, O Hiraike-Wada, T Nei, K Kawana, H Kuramoto, H Aburatani, T Yano, Y Taketani

**Affiliations:** 1 Department of Obstetrics and Gynecology, The University of Tokyo, 7-3-1 Hongo Bunkyo-ku, Tokyo 113-8655, Japan; 2 Genome Science Division, Research Center for Advanced Science and Technology, The University of Tokyo, 4-6-1 Komaba Meguro-ku, Tokyo 153-8904, Japan; 3 Department of Clinical Cytology, Kitasato University Graduate School of Medical Sciences, Kitasato 1-15-1 Sagamihara-shi, Kanagawa 228-8555, Japan

**Keywords:** *AKT1*, mutation, PI3-kinase, endometrial carcinoma

## Abstract

**Background:**

The phosphatidylinositol 3′-kinase (PI3K)–AKT pathway is activated in many human cancers and plays a key role in cell proliferation and survival. A mutation (E17K) in the pleckstrin homology domain of the *AKT1* results in constitutive AKT1 activation by means of localisation to the plasma membrane. The *AKT1* (E17K) mutation has been reported in some tumour types (breast, colorectal, ovarian and lung cancers), and it is of interest which tumour types other than those possess the *E17K* mutation.

**Methods:**

We analysed the presence of the *AKT1* (E17K) mutation in 89 endometrial cancer tissue specimens and in 12 endometrial cancer cell lines by PCR and direct sequencing.

**Results:**

We detected two *AKT1* (E17K) mutations in the tissue samples (2 out of 89) and no mutations in the cell lines. These two *AKT1* mutant tumours do not possess any mutations in *PIK3CA*, *PTEN* and *K-Ras*.

**Interpretation:**

Our results and earlier reports suggest that *AKT1* mutations might be mutually exclusive with other PI3K–AKT-activating alterations, although *PIK3CA* mutations frequently coexist with other alterations (such as *HER2*, *K-Ras* and *PTEN*) in several types of tumours.

The AKT serine/threonine kinases regulate diverse cellular processes, including cell survival, proliferation, invasion and metabolism (Vivanco and Sawyers, 2002). The phosphatidylinositol 3′-kinases (PI3Ks) are widely expressed lipid kinases that catalyse the production of the second messenger phosphatidylinositol 3,4,5-triphosphate (PIP3), which activates AKT by recruitment to the plasma membrane through direct contact of its pleckstrin homology (PH) domain (Stokoe *et al*, 1997; Lemmon and Ferguson, 2000). Constitutive PI3K–AKT pathway activation can result from various types of alterations in this pathway, including mutation or amplification of receptor tyrosine kinases (such as *EGFR* and *HER2*), mutation of *Ras*, mutation or amplification of *PIK3CA* (the p110*α* catalytic subunit of PI3K) and inactivation of the tumour suppressor gene, *PTEN* (Yuan and Cantley, 2008). In addition to amplifications in multiple AKT isoforms in pancreatic, ovarian and head and neck cancers (Engelman *et al*, 2006), a somatic missense mutation in the PH domain of *AKT1* (E17K) was identified in breast, colorectal, ovarian and lung cancers and in melanoma (Carpten *et al*, 2007; Bleeker *et al*, 2008; Davies *et al*, 2008; Malanga *et al*, 2008). However, the *AKT1* mutation has not been identified in hepatocellular, gastric and pancreatic cancers, leukemia, as well as in glioblastoma multiforme (Bleeker *et al*, 2008; Cao *et al*, 2008; Kim *et al*, 2008; Mahmoud *et al*, 2008; Mohamedali *et al*, 2008; Riener *et al*, 2008; Zenz *et al*, 2008). Further study is required to fully understand which tumour types take advantage of *Akt1* (E17K) mutations to activate the PI3K–AKT pathway.

We reported earlier that *PIK3CA* mutations frequently coexist with other PI3K-activating alterations in breast (with *HER2* and *HER3*) and endometrial cancers (with *PTEN* and *K-Ras*), and that mutant p110*α* combined with mutant Ras efficiently transformed immortalised human mammary epithelial cells (Oda *et al*, 2005, 2008). Frequent overlapping mutations of *K-Ras* and *PIK3CA* were also reported in colorectal cancer (Parsons *et al*, 2005). Although coexistent mutations of *AKT1* and *PIK3CA* mutations are suggested to be infrequent in breast cancer (Carpten *et al*, 2007; Bleeker *et al*, 2008), it remains to be elucidated whether *AKT1* mutations are mutually exclusive with all the other PI3K–AKT-activating alterations in various tumour types.

Endometrial cancer is one of the tumour types in which the PI3K–AKT pathway is frequently activated by alterations of various genes. The frequency of mutations for *PTEN*, *PIK3CA* and *K-Ras* in endometrial cancer is reported as 54, 28 and 11%, respectively (Yuan and Cantley, 2008). In this study, we screened 89 endometrial carcinoma specimens and 12 endometrial carcinoma cell lines for mutations in *Akt1* (E17K) and analysed whether *AKT1* mutations coexist with any mutations in *PTEN*, *PIK3CA* and *K-Ras*.

## Materials and methods

### Tumour samples and genomic DNA

Surgical samples were obtained from 89 patients with primary endometrial carcinomas who underwent resection of their tumours at the University of Tokyo Hospital. All patients provided informed consent for the research use of their samples and the collection, and the use of tissues for this study was approved by the appropriate institutional ethics committees. Genomic DNA was extracted by a standard SDS-proteinase K procedure. Patient characteristics (histology, tumour grade and stage) are available in Supplementary Table 1. A detailed distribution of the histological subtypes was as follows; 81 (90%) endometrioid adenocarcinomas, 3 adenosquamous carcinomas, 1 clear cell carcinoma, 1 squamous cell carcinoma and 3 mixed carcinomas.

### PCR and sequencing

The primer sequences and PCR conditions of exon 4 of the *AKT1* gene are forward: 5′-CACACCCAGTTCCTGCCT G-3′ and reverse: 5′-CCTGGTGGGCAAAGAGGGCT-3′. The PCR amplifications were with denaturation at 94°C for 5 min, followed by 35 cycles of 94°C for 30 s, 55°C for 30 s, 72°C for 60 s and final extension at 72°C for 10 min. The PCR conditions and the PCR primers for *PIK3CA* (exons 9 and 20), *PTEN* (exons 1–9) and *K-Ras* (exons 1 and 2) were described earlier (Minaguchi *et al*, 2001; Samuels *et al*, 2004; Oda *et al*, 2008). The PCR products were sequenced using the BigDye (Applied Biosystems, Foster City, CA, USA) terminator method on an autosequencer.

### Cell lines

In this study, AN3CA, KLE, HEC-1B and RL95-2 were obtained from the American Type Culture Collection (Manassas, VA, USA) and HHUA was obtained from the RIKEN CELL BANK (Tsukuba, Japan). Ishikawa3-H-12 was a generous gift from Dr Masato Nishida (Kasumigaura Medical Center, Ibaraki, Japan). HEC-6, HEC-50B, HEC-59, HEC-88, HEC-108 and HEC-116 cell lines were also analysed in this study. The culture condition of all these cell lines was described earlier (Oda *et al*, 2008).

### DNA methylation analysis

Bisulphite treatment was performed using the EZ DNA methylation kit (Zymo Research, Orange, CA, USA). As described earlier (Ehrich *et al*, 2006), we used Sequenom's MassARRAY platform to perform quantitative methylation analysis of multiple CpG sites for *PTEN* in 53 endometrial tumour specimens (Sequenom, San Diego, CA, USA). Chromosomal localisation of CpG islands for *PTEN* and the primer sequences in this study are shown in Supplementary Figure 1.

### Immunohistochemistry (IHC)

Immunohistochemistry for PTEN on 4-*μ*m tissue sections was performed and evaluated as described earlier (Minaguchi *et al*, 2007). In this study, the anti-PTEN Rabbit monoclonal antibody (138G6) (Cell Signaling, Beverly, MA, USA) was applied at a dilution of 1 : 100.

### Single nucleotide polymorphism (SNP) array

Single nucleotide polymorphism array was performed in the two *AKT1* mutant tumours with tumour DNA. Experimental procedures for GeneChip were performed according to GeneChip Expression Analysis Technical Manual (Affymetrix, Santa Clara, CA, USA), using a Human mapping 50K Array Xba I (Affymetrix).

## Results and discussions

**Figure 1:**
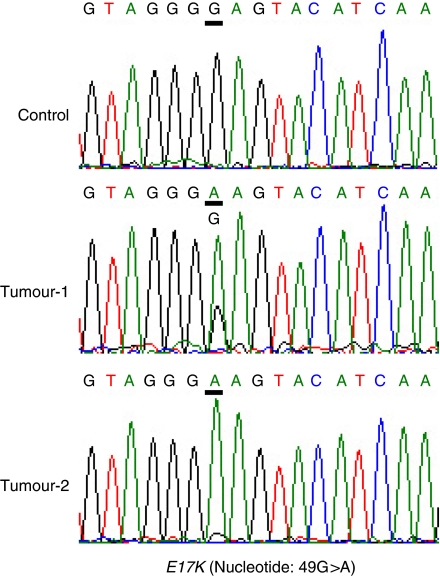
The sequence traces of two tumours and a normal control for exon 4 of *AKT1*. The *E17K* mutation is caused by a missense mutation (G to A) indicated. In tumour-2, the level of the mutant band (A) is much higher than that of the wild-type band (G). It is possible that this weak band is derived from DNA of normal cells and that the tumour might lose one allele at this locus.

The sequencing analysis for exon 4 of the *AKT1* gene in 89 tumour tissue samples of endometrial carcinomas showed the point mutation of G to A at nucleotide 49 (E17K) in two tissue samples (2.2%) (Figure 1). Both of the tumours were well-differentiated endometrioid adenocarcinomas with positive oestrogen receptor and progesterone receptor, suggesting that these two tumours are oestrogen dependent (corresponded to type I endometrial cancer). No mutations were detected in the 12 endometrial cancer cell lines.

Thereafter, we attempted to figure out the exclusivity of *AKT1* mutations and other PI3K–AKT-activating mutations (Supplementary Table 1). The genotypic pattern of the four genes (*PTEN*, *PIK3CA*, *K-Ras* and *AKT1*) in 97 endometrial carcinomas (85 tumour tissue samples and 12 cell lines) was shown in Table 1. Coexistence with other mutations is frequently observed in the *PIK3CA* mutant (28 of 34; 82%) and in the *K-Ras* mutant (13 out of 17; 76%) tumours, but the two *AKT1* mutant tumours do not possess any mutations in *PTEN*, *PIK3CA* and *K-Ras*. As PI3K and PTEN are competitive for PIP3 production, the *PIK3CA* mutation might require another upstream input or PTEN loss itself to fully activate the PI3K–AKT pathway. As AKT1 (E17K) functions downstream of PTEN and shows constitutive localisation to the plasma membrane in the absence of serum stimulation (Carpten *et al*, 2007), mutant *AKT1* (E17K) alone might be sufficient for complete activation of this pathway.

We also analysed DNA methylation and protein expression of PTEN, as hypermethylation and loss of heterozygosity (LOH) are other mechanisms to inactivate PTEN (Teng *et al*, 1997; Blanco-Aparicio *et al*, 2007). Quantitative analysis of DNA methylation using Sequenom's MassARRAY platform did not find promoter hypermethylation of *PTEN* in all the 53 samples that were examined (Supplementary Figure 2 and Supplementary Table 2), including the two *AKT1* mutant tumours. Although *PTEN* methylation had been reported in 18% of endometrial carcinomas (Salvesen *et al*, 2001), Zysman *et al* (2002) suggested that the pseudogene on chromosome 9 (Genbank accession number: AF040103), not *PTEN*, is predominantly methylated in endometrial carcinomas. In IHC, both tumours with the *AKT1* mutation were stained positively for PTEN in the cytoplasm, whereas all the four tumours with multiple frameshift mutations in *PTEN* were stained negatively (Supplementary Figure 3). We evaluated the chromosomal imbalances in the two *AKT1* mutant tumours, using SNP array (with more than 50 000 SNPs). Single nucleotide polymorphism array analysis showed that the two *AKT1* mutant tumours do not show copy number changes in the locus of *PTEN* (10q23.1) (data not shown). These data also support the fact that *AKT1* mutations are mutually exclusive with PTEN inactivation.

We found multiple *PTEN* mutations in 13 out of 85 clinical specimens and in 8 out of 12 endometrial cell lines (Supplementary Table 1), whereas LOH of *PTEN* was reported approximately at 30% in endometrial carcinomas (Toda *et al*, 2001). Thus, biallelic *PTEN* inactivation might be achieved through either biallelic mutations or monoallelic mutation with LOH in endometrial carcinomas. Considering the correlation between *PTEN* mutations and microsatellite instability (MSI) in endometrial carcinomas (Bilbao *et al*, 2006), it would be of interest to analyse whether *AKT1* and the other mutations in the PI3K pathway genes are also associated with MSI.

To date, *AKT1* (E17K) mutations have been reported in breast (25 out of 427; 5.9%), colorectal (4 out of 243; 1.6%), lung (4 out of 636; 0.6%) and ovarian cancers (1 out of 130; 0.8%) and in melanoma (1 out of 202; 0.5%). Breast, colorectal and endometrial cancers are the tumour types that frequently possess *PIK3CA* mutations (Campbell *et al*, 2004; Samuels *et al*, 2004; Oda *et al*, 2005). In lung cancer, the *AKT1* mutation was detected only in squamous cell carcinomas and not in any adenocarcinomas, which is in agreement with the higher incidence of *PIK3CA* mutations or amplifications in squamous cell carcinomas than adenocarcinomas (Kawano *et al*, 2006, 2007; Malanga *et al*, 2008). These data suggest that the *AKT1* mutation might occur in a tissue-specific manner and is more associated with the tumour types with frequent *PIK3CA* alterations.

## Figures and Tables

**Table 1:** PI3K–AKT activating mutations and their coexistence in 97 endometrial cancers

	***n* (%)**
Wild-type	24 (25)
*AKT1* mutation alone	2 (2)
*K-Ras* mutation alone	4 (4)
*PIK3CA* mutation alone	6 (6)
*PTEN* mutation alone	30 (31)
Double mutations of *K-Ras* and *PIK3CA* (w/o *PTEN* mutation)	2 (2)
Double mutations of *K-Ras* and *PTEN* (w/o *PIK3CA* mutation)	3 (3)
Double mutations of *PIK3CA* and *PTEN* (w/o *K-Ras* mutation)	18 (19)
Triple mutations of *K-Ras*, *PIK3CA* and *PTEN*	8 (8)

PI3K, phosphatidylinositol 3′-kinase.

Wild-type, no mutations in *PTEN*, *PIK3CA*, *K-Ras* and *AKT1.*

## References

[bib1] Bilbao C, Rodriguez G, Ramirez R, Falcon O, Leon L, Chirino R, Rivero JF, Falcon Jr O, Diaz Chico BN, Diaz Chico JC, Perucho M (2006) The relationship between microsatellite instability and PTEN gene mutations in endometrial cancer. Int J Cancer 119: 563–570 16506206 10.1002/ijc.21862

[bib2] Blanco-Aparicio C, Renner O, Leal JF, Carnero A (2007) PTEN, more than the AKT pathway. Carcinogenesis 28: 1379–1386 17341655 10.1093/carcin/bgm052

[bib3] Bleeker FE, Felicioni L, Buttitta F, Lamba S, Cardone L, Rodolfo M, Scarpa A, Leenstra S, Frattini M, Barbareschi M, Grammastro MD, Sciarrotta MG, Zanon C, Marchetti A, Bardelli A (2008) AKT1(E17K) in human solid tumours. Oncogene 27: 5648–5650 18504432 10.1038/onc.2008.170

[bib4] Campbell IG, Russell SE, Choong DY, Montgomery KG, Ciavarella ML, Hooi CS, Cristiano BE, Pearson RB, Phillips WA (2004) Mutation of the PIK3CA gene in ovarian and breast cancer. Cancer Res 64: 7678–7681 15520168 10.1158/0008-5472.CAN-04-2933

[bib5] Cao Z, Song JH, Kim CJ, Cho YG, Kim SY, Nam SW, Lee JY, Park WS (2008) Absence of E17K mutation in the pleckstrin homology domain of AKT1 in gastrointestinal and liver cancers in the Korean population. APMIS 116: 530–533 18754328 10.1111/j.1600-0463.2008.00998.x

[bib6] Carpten JD, Faber AL, Horn C, Donoho GP, Briggs SL, Robbins CM, Hostetter G, Boguslawski S, Moses TY, Savage S, Uhlik M, Lin A, Du J, Qian YW, Zeckner DJ, Tucker Kellogg G, Touchman J, Patel K, Mousses S, Bittner M, Schevitz R, Lai MH, Blanchard KL, Thomas JE (2007) A transforming mutation in the pleckstrin homology domain of AKT1 in cancer. Nature 448: 439–444 17611497 10.1038/nature05933

[bib7] Davies MA, Stemke Hale K, Tellez C, Calderone TL, Deng W, Prieto VG, Lazar AJ, Gershenwald JE, Mills GB (2008) A novel AKT3 mutation in melanoma tumours and cell lines. Br J Cancer 99: 1265–1268 18813315 10.1038/sj.bjc.6604637 PMC2570525

[bib8] Ehrich M, Field JK, Liloglou T, Xinarianos G, Oeth P, Nelson MR, Cantor CR, van den Boom D (2006) Cytosine methylation profiles as a molecular marker in non-small cell lung cancer. Cancer Res 66: 10911–10918 17108128 10.1158/0008-5472.CAN-06-0400

[bib9] Engelman JA, Luo J, Cantley LC (2006) The evolution of phosphatidylinositol 3-kinases as regulators of growth and metabolism. Nat Rev Genet 7: 606–619 16847462 10.1038/nrg1879

[bib10] Kawano O, Sasaki H, Endo K, Suzuki E, Haneda H, Yukiue H, Kobayashi Y, Yano M, Fujii Y (2006) PIK3CA mutation status in Japanese lung cancer patients. Lung Cancer 54: 209–215 16930767 10.1016/j.lungcan.2006.07.006

[bib11] Kawano O, Sasaki H, Okuda K, Yukiue H, Yokoyama T, Yano M, Fujii Y (2007) PIK3CA gene amplification in Japanese non-small cell lung cancer. Lung Cancer 58: 159–160 17681398 10.1016/j.lungcan.2007.06.020

[bib12] Kim MS, Jeong EG, Yoo NJ, Lee SH (2008) Mutational analysis of oncogenic AKT E17K mutation in common solid cancers and acute leukaemias. Br J Cancer 98: 1533–1535 18392055 10.1038/sj.bjc.6604212 PMC2391109

[bib13] Lemmon MA, Ferguson KM (2000) Signal-dependent membrane targeting by pleckstrin homology (PH) domains. Biochem J 350(Pt 1): 1–18 10926821 PMC1221219

[bib14] Mahmoud IS, Sughayer MA, Mohammad HA, Awidi AS, EL-Khateeb MS, Ismail SI (2008) The transforming mutation E17K/AKT1 is not a major event in B-cell-derived lymphoid leukaemias. Br J Cancer 99: 488–490 18665177 10.1038/sj.bjc.6604512 PMC2527790

[bib15] Malanga D, Scrima M, De Marco C, Fabiani F, De Rosa N, De Gisi S, Malara N, Savino R, Rocco G, Chiappetta G, Franco R, Tirino V, Pirozzi G, Viglietto G (2008) Activating E17K mutation in the gene encoding the protein kinase AKT1 in a subset of squamous cell carcinoma of the lung. Cell Cycle 7: 665–669 18256540 10.4161/cc.7.5.5485

[bib16] Minaguchi T, Nakagawa S, Takazawa Y, Nei T, Horie K, Fujiwara T, Osuga Y, Yasugi T, Kugu K, Yano T, Yoshikawa H, Taketani Y (2007) Combined phospho-Akt and PTEN expressions associated with post-treatment hysterectomy after conservative progestin therapy in complex atypical hyperplasia and stage Ia, G1 adenocarcinoma of the endometrium. Cancer Lett 248: 112–122 16919866 10.1016/j.canlet.2006.06.013

[bib17] Minaguchi T, Yoshikawa H, Oda K, Ishino T, Yasugi T, Onda T, Nakagawa S, Matsumoto K, Kawana K, Taketani Y (2001) PTEN mutation located only outside exons 5, 6, and 7 is an independent predictor of favorable survival in endometrial carcinomas. Clin Cancer Res 7: 2636–2642 11555573

[bib18] Mohamedali A, Lea NC, Feakins RM, Raj K, Mufti GJ, Kocher HM (2008) AKT1 (E17K) mutation in pancreatic cancer. Technol Cancer Res Treat 7: 407–408 18783292 10.1177/153303460800700509

[bib19] Oda K, Okada J, Timmerman L, Rodriguez Viciana P, Stokoe D, Shoji K, Taketani Y, Kuramoto H, Knight ZA, Shokat KM, McCormick F (2008) PIK3CA cooperates with other phosphatidylinositol 3′-kinase pathway mutations to effect oncogenic transformation. Cancer Res 68: 8127–8136 18829572 10.1158/0008-5472.CAN-08-0755

[bib20] Oda K, Stokoe D, Taketani Y, McCormick F (2005) High frequency of coexistent mutations of PIK3CA and PTEN genes in endometrial carcinoma. Cancer Res 65: 10669–10673 16322209 10.1158/0008-5472.CAN-05-2620

[bib21] Parsons DW, Wang TL, Samuels Y, Bardelli A, Cummins JM, DeLong L, Silliman N, Ptak J, Szabo S, Willson JK, Markowitz S, Kinzler KW, Vogelstein B, Lengauer C, Velculescu VE (2005) Colorectal cancer: mutations in a signalling pathway. Nature 436: 792 16094359 10.1038/436792a

[bib22] Riener MO, Bawohl M, Clavien PA, Jochum W (2008) Analysis of oncogenic AKT1 p.E17K mutation in carcinomas of the biliary tract and liver. Br J Cancer 99: 836 18728674 10.1038/sj.bjc.6604498 PMC2528140

[bib23] Salvesen HB, MacDonald N, Ryan A, Jacobs IJ, Lynch ED, Akslen LA, Das S (2001) PTEN methylation is associated with advanced stage and microsatellite instability in endometrial carcinoma. Int J Cancer 91: 22–26 11149415 10.1002/1097-0215(20010101)91:1<22::aid-ijc1002>3.0.co;2-s

[bib24] Samuels Y, Wang Z, Bardelli A, Silliman N, Ptak J, Szabo S, Yan H, Gazdar A, Powell SM, Riggins GJ, Willson JK, Markowitz S, Kinzler KW, Vogelstein B, Velculescu VE (2004) High frequency of mutations of the PIK3CA gene in human cancers. Science 304: 554 15016963 10.1126/science.1096502

[bib25] Stokoe D, Stephens LR, Copeland T, Gaffney PR, Reese CB, Painter GF, Holmes AB, McCormick F, Hawkins PT (1997) Dual role of phosphatidylinositol-3,4,5-trisphosphate in the activation of protein kinase B. Science 277: 567–570 9228007 10.1126/science.277.5325.567

[bib26] Teng DH, Hu R, Lin H, Davis T, Iliev D, Frye C, Swedlund B, Hansen KL, Vinson VL, Gumpper KL, Ellis L, El Naggar A, Frazier M, Jasser S, Langford LA, Lee J, Mills GB, Pershouse MA, Pollack RE, Tornos C, Troncoso P, Yung WK, Fujii G, Berson A, Steck PA, Bookstein R, Bolen JB, Tavtigian SV (1997) MMAC1/PTEN mutations in primary tumor specimens and tumor cell lines. Cancer Res 57: 5221–5225 9393738

[bib27] Toda T, Oku H, Khaskhely NM, Moromizato H, Ono I, Murata T (2001) Analysis of microsatellite instability and loss of heterozygosity in uterine endometrial adenocarcinoma. Cancer Genet Cytogenet 126: 120–127 11376804 10.1016/s0165-4608(00)00400-3

[bib28] Vivanco I, Sawyers CL (2002) The phosphatidylinositol 3-Kinase AKT pathway in human cancer. Nat Rev Cancer 2: 489–501 12094235 10.1038/nrc839

[bib29] Yuan TL, Cantley LC (2008) PI3K pathway alterations in cancer: variations on a theme. Oncogene 27: 5497–5510 18794884 10.1038/onc.2008.245 PMC3398461

[bib30] Zenz T, Dohner K, Denzel T, Dohner H, Stilgenbauer S, Bullinger L (2008) Chronic lymphocytic leukaemia and acute myeloid leukaemia are not associated with AKT1 pleckstrin homology domain (E17K) mutations. Br J Haematol 141: 742–743 18410456 10.1111/j.1365-2141.2008.07113.x

[bib31] Zysman MA, Chapman WB, Bapat B (2002) Considerations when analyzing the methylation status of PTEN tumor suppressor gene. Am J Pathol 160: 795–800 11891178 10.1016/S0002-9440(10)64902-4 PMC1867163

