# Supplementary material for: The oncogenic mutation in the pleckstrin homology domain of AKT1 in endometrial carcinomas
Source: Br J Cancer. 2009 Jun 2;101(1):145–8. doi: 10.1038/sj.bjc.6605109 (PMC2713716; doi:10.1038/sj.bjc.6605109)
Supplement: Supplementary Figure 1 [file 6605109x1.ppt]

## Slide 1
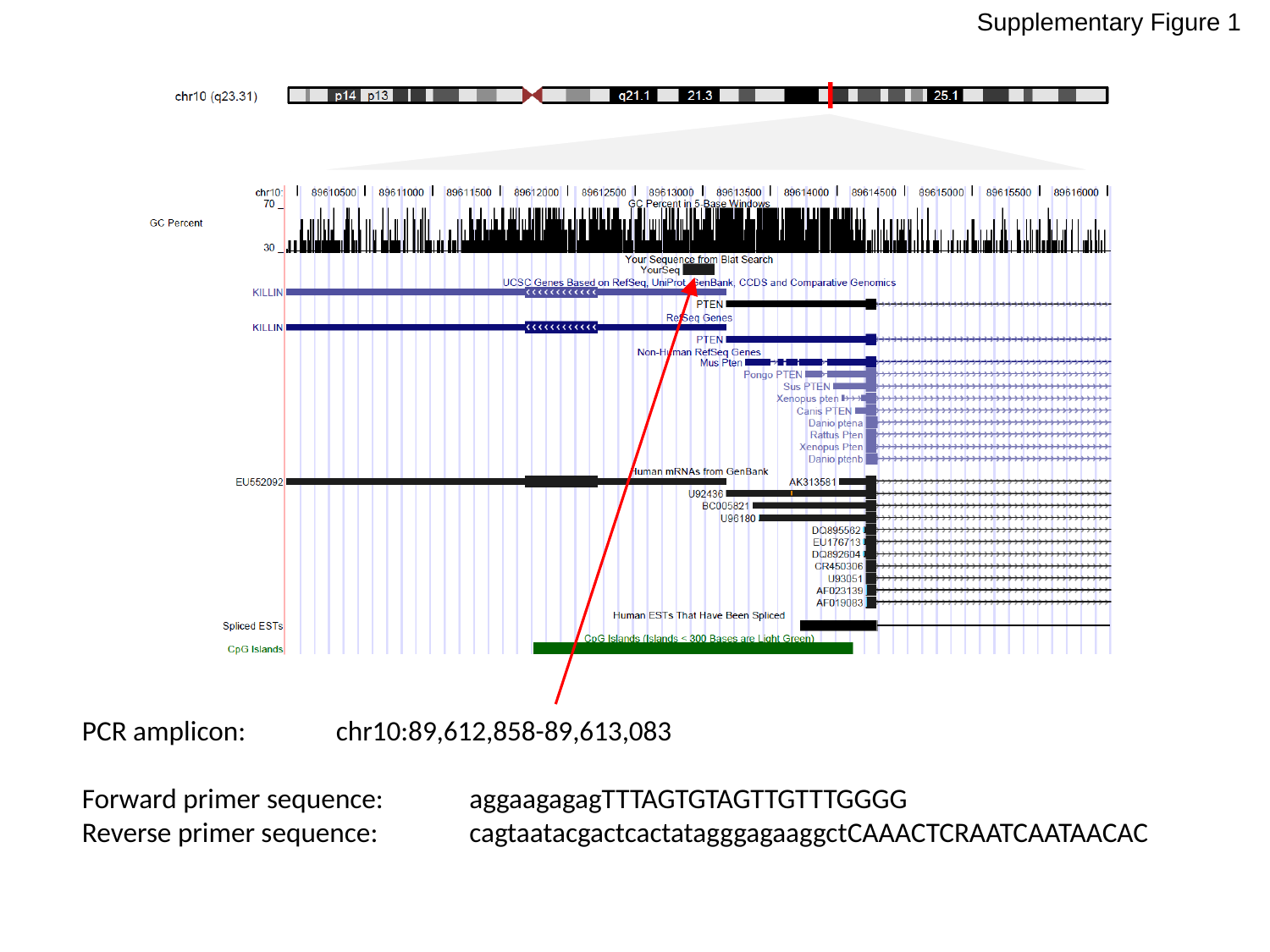

Supplementary Figure 1
PCR amplicon:	chr10:89,612,858-89,613,083
Forward primer sequence:	 aggaagagagTTTAGTGTAGTTGTTTGGGG
Reverse primer sequence:	 cagtaatacgactcactatagggagaaggctCAAACTCRAATCAATAACAC
