# Supplementary figures and images for: The oncogenic mutation in the pleckstrin homology domain of AKT1 in endometrial carcinomas
Source: Br J Cancer. 2009 Jun 2;101(1):145–8. doi: 10.1038/sj.bjc.6605109 (PMC2713716; doi:10.1038/sj.bjc.6605109)

## Slide 1
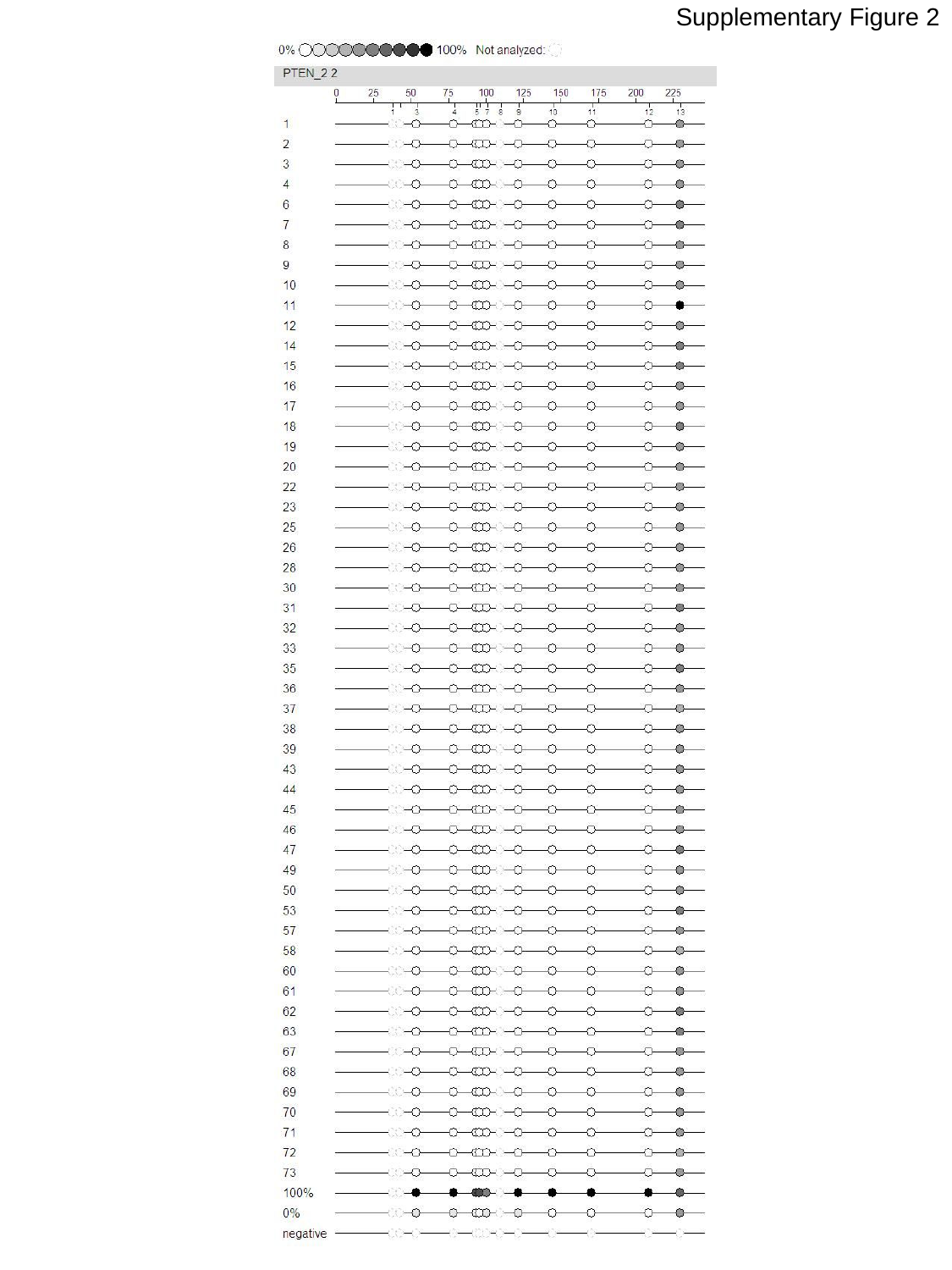

Supplementary Figure 2

Supplement: Supplementary Figure 2 [file 6605109x2.ppt]

## Slide 1
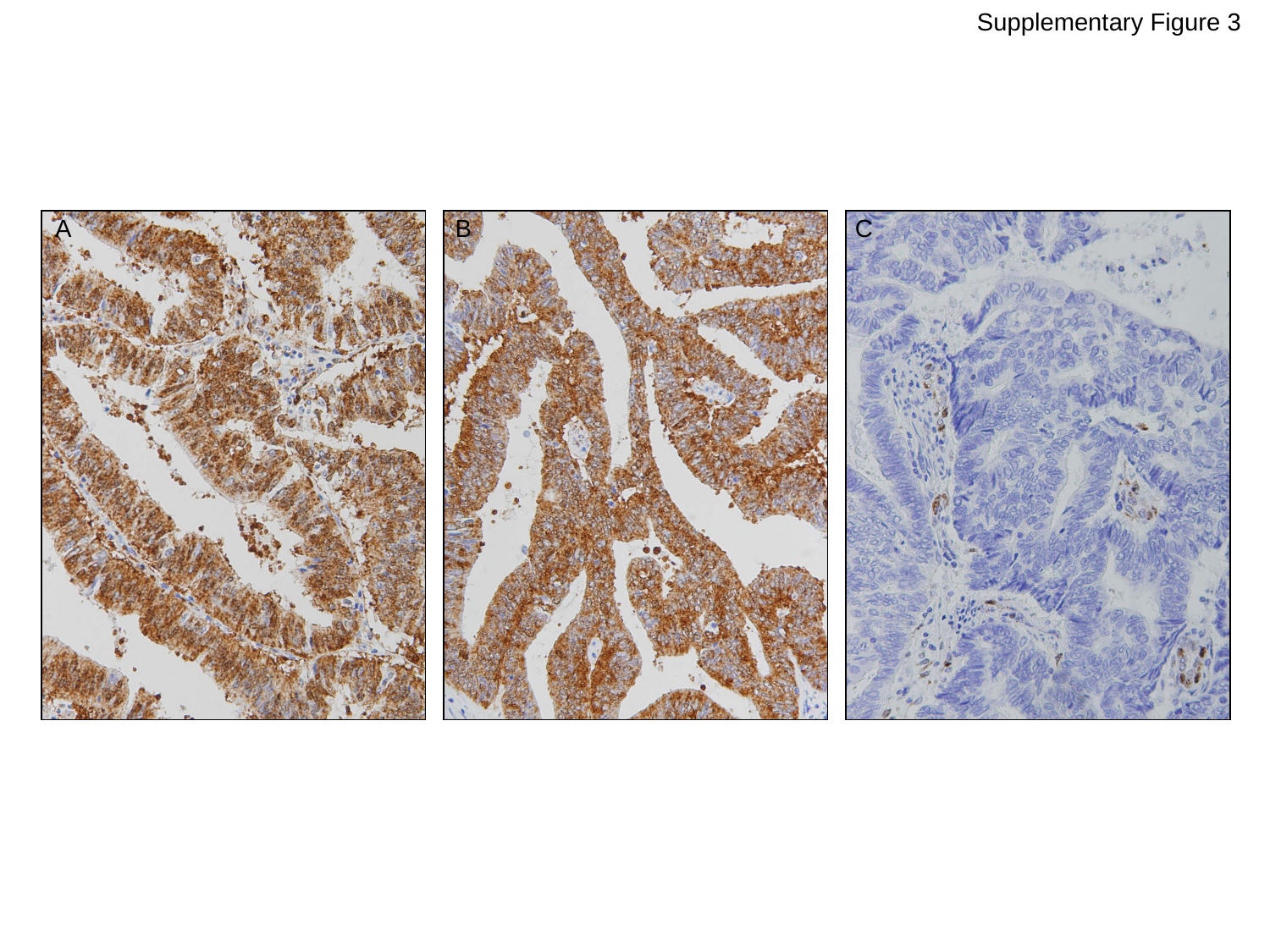

Supplementary Figure 3
A
B
C

Supplement: Supplementary Figure 3 [file 6605109x3.ppt]
