# Supplementary material for: The oncogenic mutation in the pleckstrin homology domain of AKT1 in endometrial carcinomas
Source: Br J Cancer. 2009 Jun 2;101(1):145–8. doi: 10.1038/sj.bjc.6605109 (PMC2713716; doi:10.1038/sj.bjc.6605109)
Supplement: Supplementary Figure Legends [file 6605109x4.doc]

**Supplementary Figure Legends**

**Supplementary Figure 1.**

**Chromosomal mapping of CpG islands for *PTEN* and PCR amplicon for *PTEN* methylation analysis.**

GC percent in 5-base windows in chromosome 10: 89,601,500-89,616,000, transcription start site of *PTEN* and primer sequences for the PCR amplicon are presented.

**Supplementary Figure 2.**

***PTEN* methylation analysis in 53 endometrial cancer specimens.**

CpG sites (from #1 to #13) were analyzed for methylation. No significant methylation were observed at CpG # 3,4, 5.6, 7, 9, 10, 11 and 12 in all specimens. The raw data is listed in Supplementary Table 2.

**Supplementary Figure 3.**

**PTEN protein expression by Immunohistochemistry (IHC).**

Normal PTEN expression in cytoplasm is observed in patients A (#10) and B (#17), both of which possess *AKT1* (E17K) mutation. PTEN expression is absent in patient C (#43) with multiple frameshift mutations in *PTEN*.
